# Evaluation of the UV Protection Properties of Para-Aramid Woven Fabrics with Various Specialty Core Yarns

**DOI:** 10.3390/polym16213090

**Published:** 2024-10-31

**Authors:** Klara Kostajnšek, Matejka Bizjak, Gözde Ertekin, Mustafa Ertekin

**Affiliations:** 1 Department of Textiles, Graphics Art and Design, Faculty for Natural Sciences and Engineering, University of Ljubljana, Snežniška 5, 1000 Ljubljana, Slovenia; matejka.bizjak@ntf.uni-lj.si; 2 Department of Textile Engineering, Faculty of Engineering, Ege University, 35100 Izmir, Turkey; gozde.damci@ege.edu.tr (G.E.); mustafa.ertekin@ege.edu.tr (M.E.)

**Keywords:** para-aramid fibers, core-spun yarns, ultraviolet protection factor (UPF), UV resistance, protective textiles, material degradation

## Abstract

Para-aramid fibers, known for their remarkable strength and thermal stability, are frequently employed in protective textiles for military and aerospace applications. However, continuous exposure to ultraviolet (UV) radiation can damage their protective characteristics. This study analyzes the ultraviolet protection factor (UPF) and UV transmittance of woven fabrics produced from 30/2 Ne spun para-aramid yarns in the warp and 10 Ne core-spun yarns in the weft. The weft yarns consisted of three sheath fibers—para-aramid, meta-aramid, and polyester—in combination with different specialty core materials. The results show significant differences in UPF before and after UV exposure, with para-aramid sheaths giving the highest improvement. UV exposure caused structural changes in the fibers, resulting in increased UV protection, particularly in fabrics with para-aramid sheaths. This study concludes that the combination of para-aramid fibers with specific core materials significantly enhances UV protection, making them well-suited for applications in high UV exposure environments.

## 1. Introduction

In recent decades, protection from harmful UV radiation has become increasingly important due to the thinning of the ozone layer in the atmosphere and exposure to artificial sources. Depending on the impact on human health and the environment, UV radiation is divided into three ranges of different intensities and wavelength: the long-wave ultraviolet A (UVA) or black light range (315–400 nm), the medium-wave ultraviolet B (UVB) range (280–315 nm), and the short-wave or bactericidal ultraviolet C (UVC) range (100–280 nm). Considering the increasing importance of UV protection, especially concerning ozone depletion and artificial UV sources, the focus has moved on to UVA and UVB radiation, since UVC is absorbed by the Earth’s atmosphere. UVA rays penetrate deeper into the skin and cause long-term damage [1], while UVB rays are known for causing superficial skin burns [2]. The efficiency of textiles in blocking UV radiation is quantified by the Ultraviolet Protection Factor (UPF) which indicates the effectiveness of a fabric at protecting human skin against ultraviolet radiation through the fabric. UPF is calculated based on the ratio of UV radiation transmitted through the fabric to the amount of UV radiation incident on the fabric, with both UVA and UVB rays taken into account. Higher UPF ratings indicate better protection, as they allow less UV radiation to pass through the fabric. A fabric with a UPF rating of 50, for instance, allows only 2% of UV rays to penetrate, making it highly effective in UV protection.

Various technical textile products, such as sunshades and curtains, have emerged as highly effective solutions for protection and they play a crucial role in shielding individuals by preventing UV radiation from reaching them [3,4].

Aramid fibers, known for their superior strength, durability, and heat resistance, have been widely used in the production of protective textiles. In particular, para-aramid fibers, such as Kevlar^®^, are critical components in sectors that require high durability and resistance to environmental factors, including ultraviolet (UV) radiation. Such resistance is of primary importance for the sustained functionality and performance of materials used in military and aerospace applications. Nevertheless, despite their inherent strength, aramid fibers are susceptible to degradation when exposed to prolonged UV radiation, which significantly reduces their strength and limits their functional lifespan [5,6,7]. Para-aramid fabrics with UPF resistance are particularly valuable in environments with prolonged UV exposure, as they retain their protective qualities over time.

A review of previous studies reveals that numerous researchers have investigated various surface modification and coating techniques [8,9,10,11,12] and conducted optimization studies [13,14,15,16] to enhance the UV resistance of aramid fibers without compromising their mechanical properties. However, research on the UV protection properties of different core–sheath combinations to assess their effectiveness in improving UV resistance is limited. Therefore, this study aims to evaluate the UV protection properties of plain-woven fabrics made from 30/2 Ne spun para-aramid warp yarns and 10/1 Ne core-spun weft yarns. The weft yarns incorporate three different sheath fibers—para-aramid, me-ta-aramid, and polyester Trevira CS—and seven different core materials, including E-glass, polyester (PES), high-tenacity polyamide (PA HT), polypropylene (PP), high-tenacity polyester (PES HT), Technora T240, and Dyneema SK75.

The main objective of this study is to investigate how different core and sheath yarn combinations affect UPF and UV transmittance (UVA and UVB), and how these properties change after exposure to daylight, determining their suitability for protective textiles in high UV exposure environments.

## 2. Materials and Methods

### 2.1. Materials

**Figure 1:**
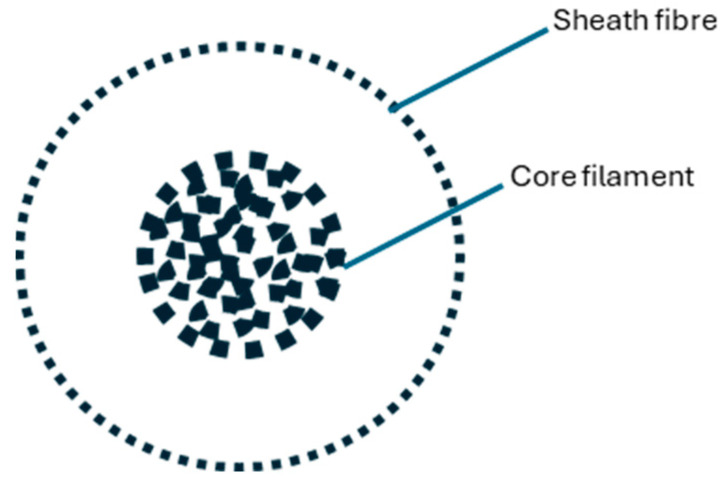
Cross-section of core-spun yarn.

To produce the fabrics analyzed in this study, the yarn was carefully designed and produced on a Pinter Merlin spinning machine (Pinter Merlin, Biella, Italy). The warp yarn was a 30/2 Ne spun yarn produced from para-aramid fiber. The yarns used in the weft were core-spun yarns with a core to sheath ratio of 19%/81% and a final yarn count of 10/1 Ne. The core yarn spinning method, which is illustrated in Figure 1, is used to wrap different core materials within sheath fibers. This method significantly improves the functionality and performance of the yarns [17].

### 2.2. Fabric Production

**Figure 2:**
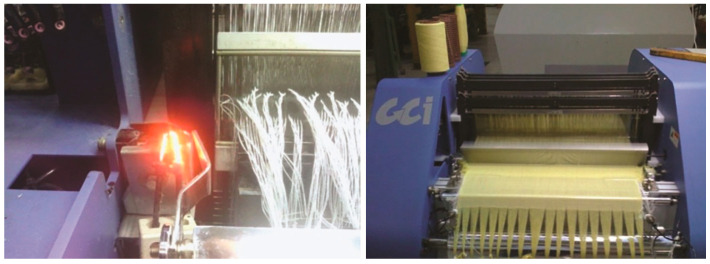
CCI SL 8900s weaving loom with a modified weft cutting system.

All samples were woven using a plain weave structure with the same warp and weft densities of 22 ends/cm and 15 picks/cm, respectively, on a CCI SL 8900s weaving loom (CCI Tech, New Taipei City, Taiwan) (Figure 2). A total of 24 fabric samples were produced, each characterized as detailed in Table 1.

### 2.3. Methods

#### 2.3.1. Measurements of Weight and Thickness

The weight of the fabric samples was measured according to the BS EN 12127:1998 standard. Weight measurements were performed with three replicates. Thickness measurements were conducted using the SDL Atlas Digital Thickness Tester (SDL Atlas, Rock Hill, SC, USA), following the EN ISO 5084 standard [18], with the results representing the averages of five readings. Fabric density was calculated by dividing fabric weight to fabric thickness.

#### 2.3.2. UV Measurements

The UV transmission and UV reflection of the samples were measured using the, UV/VIS Spectrophotometer Lambda 800, PELA-1000 (PerkinElmer, Beaconsfield, UK) according to the SIST EN 13758-1 standard [19], with the measurement of each sample repeated five times vertically and horizontally and at an angle of 45°. Data from the 400–290 nm range were used to calculate the UPF (Equation (1)), data from the 400–315 nm range were used to calculate UVA (Equation (2)), and data from the 315–290 nm range were used to calculate UVB (Equation (3)):

UPF = ∑_(400-290)_ E(λ) × ε(λ) × ∆λ/∑_(400-290)_ E(λ) × ε(λ) × T(λ) × ∆λ (1)

where E(λ) represents solar spectral radiation (Wm^−2^nm^−1^), ε(λ) is relative erythema effectiveness, T(λ) is spectral transmittance of a sample at wavelength λ(%), and Δλ a wavelength interval (nm).

UVA_i_ = 1/m ∑_(400-315)_ T_i_(λ) (2)

UVB_i_ = 1/k ∑_(315-290)_ T_i_(λ) (3)

where T_i_(λ) is the spectral transmittance of specimen i at wavelength λ, m and k are the number of measurement points between 315 nm in 400 and between 290 nm and 315 nm, respectively.

#### 2.3.3. Measurement of Light Fastness

Xenotest Alpha (Atlas Material Testing Technology, Mount Prospect, IL, USA) is a type of light fastness test that uses xenon arc weathering equipment to simulate natural daylight (Xe light) and evaluate the color fastness of textiles and any other properties that may be affected by exposure to light. These tests are crucial to determine how textiles behave over time when exposed to light, which is an important factor in assessing the quality and durability of textile products.

In this study, the focus was on determining the impact of light exposure on material properties, particularly its UV resistance. Samples were subjected to controlled conditions for 72 h (temperature: 35 °C, relative humidity: 70%, irradiance: 45 W/m^2^) following the SIST EN ISO 105-B01:2014 standard “Textiles—Tests for colour fastness Part B01: Colour fastness to light: Daylight” [20]. We performed three repetitions for each sample.

An optical method was used to measure diameter and density. The images of the examined samples were taken with a stereomicroscope (Leica S9i, Microsystems GmbH, Wetzlar, Germany) with 20× magnification, and the measurement was repeated five times on each sample. The Image J program was used to analyze the images.

#### 2.3.4. Statistical Analysis

The mean and standard deviation were calculated for each set of results. To assess the significance of differences between the samples, statistical analysis was performed using one-way ANOVA, followed by Duncan post hoc test for multiple comparisons. The statistical significance was determined at a threshold of *p* < 0.05. All statistical analyses were conducted using SPSS software (version 26.0, IBM Corporation, New York, NY, USA).

## 3. Results and Discussion

In this study, the UV protection properties of 24 fabric samples with different core and sheath combinations were investigated to determine how these combinations affect the UPF and the transmittance of UVA and UVB rays before and after daylight exposure. At the same time, measured reflection and calculated absorption values were also examined.

### 3.1. Weight and Thickness Analysis

Some studies have already shown that thicker fabrics provide better UV protection due to the higher amount of UV-blocking material [3,21,22,23,24,25]. Therefore, the weight and thickness (Table 1) of the fabric samples were crucial in determining their UV protection ability. The weight of the lightest sample made of E-glass core and para-aramid sheath was 168.04 g/m^2^, while the heaviest sample made of Dyneema SK75 core and PES Trevira CS sheath was 191.02 g/m^2^. The different weight values of the samples are influenced by the raw material used as well as the thread density of the fabric and the crimp ratio. The heavier fabrics generally had better UV-blocking properties.

### 3.2. UPF Analysis

#### 3.2.1. Initial UPF Values

The initial UPF values varied between the fabric samples (Table 2), significantly influenced by the sheath and core materials. Fabrics with a para-aramid sheath demonstrated the highest initial UPF values. For example, the sample with a para-aramid sheath and a core of high-tenacity polyamide (PA HT) exhibited a UPF of 53.63. UPF values above 40–50 (rating 50+) are considered excellent protection, i.e., they block more than 97.5% of UV radiation. This high value can be attributed to the dense molecular structure and low porosity of para-aramid fibers, which provide significant resistance to UV radiation due to their high crystallinity [26]. In addition to the UPF values, the transmittance of ultraviolet light B (UVB) and ultraviolet light A (UVA) before and after UV exposure are given in Table 2.

Transmittance refers to the amount of UV radiation (290–400 nm wavelengths) that passes through the fabric, with higher transmittance indicating lower UV protection. UPF is a measure of the UV radiation (both UVA and UVB) that is blocked by the fabric (Equation (1)) and is inversely related to transmittance; as transmittance of a fabric decreases, UPF increases [27]. The fabrics were exposed to xenon arc light at an intensity of 42 W/m^2^, simulating daylight conditions for 72 h.

Table 3 shows the p-values of the UV (before/after exposure) and UVR (before/after exposure) parameters and contains the results of the analysis of variance using Duncan’s multiple comparison.

#### 3.2.2. UPF Values After Exposure

**Figure 3:**
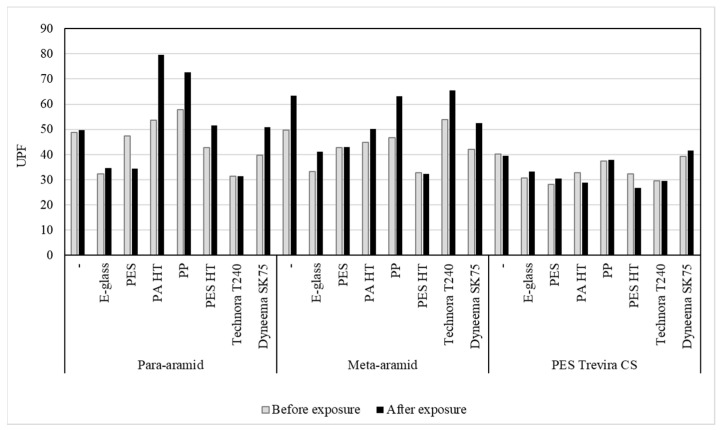
UPF comparison before and after UV exposure.

After exposure to UV radiation, most fabrics showed an increase in UPF values (Figure 3). Fabrics with a para-aramid sheath and a high-tenacity polyamide (PA HT) core, showed an increase in UPF from 53.63 to 79.53. This increase is likely due to the formation of a protective surface layer on the para-aramid fibers, enhancing their UV-blocking ability, as also reported by Dong and Jang [28].

UV exposure leads to the degradation of the outermost polymer chains in para-aramid fibers, causing surface oxidation and cross-linking. This process results in the formation of a protective layer that reduces the amount of UV radiation penetrating deeper into the fiber. This phenomenon has been observed in other high-performance fibers and is thought to improve UV protection by creating a more UV-resistant surface [29].

#### 3.2.3. Transmittance of UVB and UVA Light After Exposure

The transmittance of UVB rays was significantly reduced in fabrics with a para-aramid sheath. A para-aramid sheath with a PA HT core fabric initially had a UVB transmittance of 1.97, which decreased to 1.26 after Xe light exposure. This reduction indicates that UV exposure improves the fabric’s ability to block UVB rays, which are known for causing superficial skin burns [2]. The improvement in UV blocking after exposure can be attributed to changes in the fiber structure. As the fibers are exposed to UV light, degradation at the surface causes cross-linking and oxidation, which enhances the fabric’s ability to absorb UV radiation. The color change from yellow to brownish-yellow also increases UV absorption, as darker colors tend to block more UV light [6,30]. The PES Trevira CS samples have the highest values for the transmittance of UVB rays, which consequently has the lowest UPF values. It is well known that UPF and transmittance are directly related and essentially interdependent. The UPF is calculated based on the transmittance and it indicates how much UV radiation is blocked by the fabric (Equation (1)).

According to Table 3, the *p*-values for the effect of material type on UV (before/after exposure) properties were significant, indicating that material composition plays a key role in UV performance (*p* < 0.05). The para-aramid sheath again showed significant protection. The variance analysis presented in Table 3 shows that materials such as para-aramid exhibited significantly lower UV transmittance after exposure compared to materials like Polyester Trevira CS. This demonstrates that para-aramid-based fabrics maintain superior UV-blocking properties even after exposure to sunlight, as opposed to materials like polyester, which showed higher UV transmittance post-exposure (*p* < 0.05). Para-aramid fibers combined with a PA HT core showed superior UV-blocking performance, with a notable reduction in UV transmission after exposure (Table 3). The para-aramid sheath again showed significant protection. Para-aramid sheath with PA HT core fabric showed a decrease in UVA transmittance from 1.79 to 1.22 after exposure. Para-aramid also has the lowest UVA transmittance values compared to the other two sheath fiber materials. This improvement suggests that the protective surface layer formed during UV exposure also increases the blocking of UVA rays. This layer, which results from oxidation and cross-linking of the polymer chains, acts as an additional barrier to UV radiation, improving the fabric’s UV resistance [31]. Para-aramid fibers have a highly ordered molecular structure with high crystallinity, which makes them more effective at blocking UVA radiation. This dense molecular arrangement prevents UVA rays from penetrating the fibers as easily as they do in fibers with lower crystallinity, such as meta-aramid and polyester [16]. PES Trevira CS has the highest UVA transmittance values before and after UV irradiation at 5%, while meta-aramid and para-aramid have approximately 2.16% and 2.14%, respectively.

#### 3.2.4. Ultraviolet Light Reflection R (%) and Absorption A (%) After Exposure

Table 4 shows the results of the measured ultraviolet reflection UV R (%) and the calculated values of the ultraviolet absorption UV A (%) for the wavelength range of 290–400 nm, with mean values X and SD (for measured parameters).

**Figure 4:**
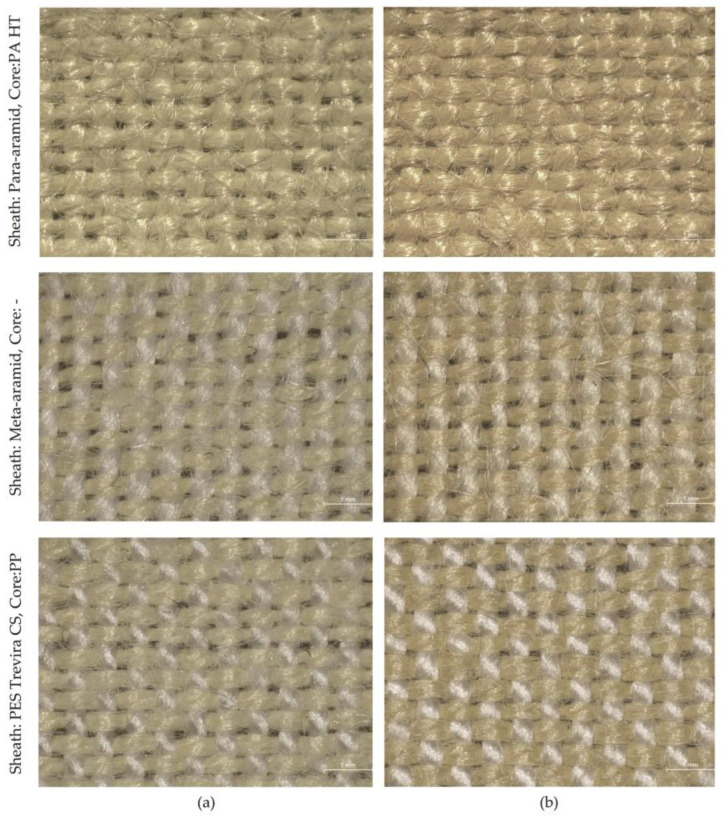
Fabric samples (**a**) before exposure, and (**b**) after exposure (Stereomicroscope Leica S9i Microsystems GmbH, Wetzlar, Germany). magnification 20×).

After exposure to Xe light, the UV reflection R (%) decreased for all samples, while the UV absorption values A (%) increased. The Duncan post hoc test highlighted that UVR values before and after UV exposure were significantly different among the tested samples (*p* < 0.05) (Table 3). As seen in Table 3, the variance analysis of UVR properties indicated that some fabric combinations, particularly those with para-aramid cores, experienced a significant reduction in UVR post-exposure compared to PES Trevira CS fabrics. The highest R values (18.52%) were found in the samples with PES Trevira CS, where the difference before and after exposure was the smallest (0.40%). The lowest reflection values were shown by samples with para-aramid, which fell by approx. 2% from 8% to 6% after exposure. The decrease in UVR after exposure to UV light highlights the structural resilience of certain fiber compositions, especially para-aramid, which likely formed a protective surface layer after exposure. The samples with the lowest UV transmittance (highest UPF values) are darker than the other samples (Figure 4). Darker colored samples have better protective properties with the same structural properties, as they absorb in the UV range and transmit less UV radiation. As absorption and/or reflection increase, transmittance decreases, resulting in a higher UPF [32,33].

Absorption was higher for all samples after exposure, highest for para-aramid (average 89.40% before and 91.51% after exposure) and lowest for PES Trevira CS (average 78.50% before and 78.80% after exposure) samples. The reflection and absorption of ultraviolet light were significantly influenced by both the type of core and sheath materials. Fabrics with para-aramid sheaths exhibited higher UV absorption, while those with polyester Trevira CS sheaths had lower absorption but higher UV reflection. These results are consistent with studies by Wakatsuki et al. [16], which highlight the importance of fiber structure in determining UV-blocking efficacy, as para-aramid fibers demonstrated increased UV absorption following exposure, contributing to improved protection.

### 3.3. Influence of the Core Material and the Sheath Fiber on the UPF

The core material significantly influenced the UPF results. The para-aramid sheath with PA HT core fabric showed the highest increase in UPF, indicating that PA HT core complements the para-aramid sheath in enhancing UV protection. The core’s compatibility and interaction with the sheath material likely contribute to this effect. The para-aramid sheath with Dyneema SK75 core also showed a significant increase in UPF from 39.68 to 50.87, highlighting the effectiveness of Dyneema as a core material. In contrast, fabrics with a polyester sheath generally had lower UPF values both before and after UV exposure, indicating the lower effectiveness of polyester in UV protection compared to para-aramid fibers. The interaction between the core and sheath materials plays a crucial role in UV protection. A compatible core material, such as PA HT, enhances the structural stability of the sheath, helping to maintain its UV-blocking properties. The core material can act as a supportive matrix, preventing excessive deformation and fiber breakage under UV exposure. This structural integrity ensures that the sheath retains its protective properties, reducing UV transmittance and increasing UPF values [29].

The type of sheath fiber used in fabric construction significantly influenced the UPF and UV results. Fabrics with para-aramid sheath fibers demonstrated the highest overall UPF values and the most considerable increases in UPF after UV exposure. This indicates that a para-aramid sheath is highly effective in enhancing UV protection. The UV resistance properties of para-aramid fibers, combined with the structural changes and color transition from yellow to brownish-yellow after exposure, contribute to this significant enhancement in UV absorption capabilities. The UV exposure leads to chemical changes on the surface of the para-aramid fibers, including oxidation and the formation of carbonyl and hydroxyl groups, which alter the molecular structure. These changes cause the fibers to become less crystalline and more amorphous, resulting in an increase in the fiber’s diameter. The color change from yellow to brownish-yellow is linked to the formation of chromophores on the fiber surface, which enhances the absorption of UV light by increasing the material’s UV absorbance [6,30,34].

Meta-aramid sheath fibers also showed improved UPF values after exposure, but their effectiveness was less noticeable compared to para-aramid sheath. The structural and chemical properties of meta-aramid fibers contribute to their UV-blocking ability, although not as much as para-aramid fibers. In contrast, fabrics with the polyester Trevira CS sheath fiber exhibited the lowest UPF values, both before and after UV exposure. This highlights relatively lower effectiveness of polyester in UV protection due to its lower UV resistance [35] compared to aramid fibers. Meta-aramid fibers have a less ordered molecular structure compared to para-aramid fibers, resulting in lower crystallinity. This difference in crystallinity affects their UV-blocking ability, as the less dense molecular arrangement in meta-aramid fibers allows more UV radiation to penetrate the material. Additionally, meta-aramid fibers have lower tensile strength and UV resistance, making them more susceptible to degradation under prolonged UV exposure. In contrast, the high crystallinity of para-aramid fibers provides superior UV resistance, as the tightly packed molecular chains create a more effective barrier against UV radiation [6,36].

### 3.4. Structural Changes After UV Exposure

**Figure 5:**
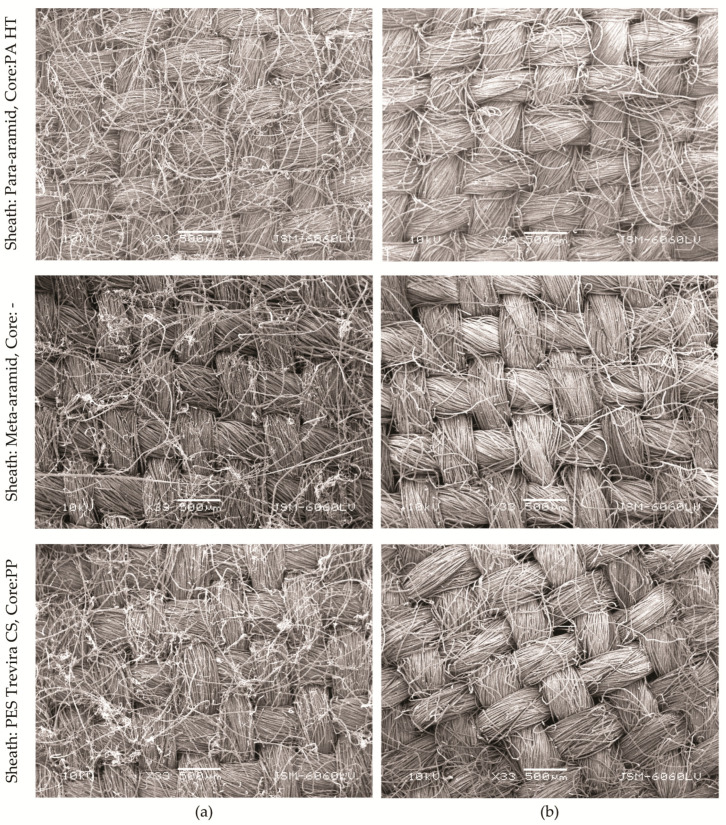
SEM images of fabric samples (**a**) before exposure, and (**b**) after exposure.

Scanning electron microscopy (SEM) was used for microscopic surface analysis, which revealed that UV exposure significantly affects the surface properties of aramid fabrics, resulting in smoother, less “hairy” surfaces (Figure 5). The surface of the spun aramid fabrics appeared hairier before UV exposure, becoming smoother after exposure. This reduction in surface irregularities is likely to contribute to the increased UPF, as a more uniform surface better blocks UV radiation. This phenomenon is due to degradation and oxidation processes at the fiber surface when exposed to UV radiation. A more uniform surface reduces the number of pores and irregularities in the fabric, which could allow UV radiation to pass through. After UV exposure, the surface of the para-aramid fibers becomes smoother due to the degradation of loose fibers and the formation of a denser protective layer. This reduction in surface irregularities leads to fewer pathways for UV radiation to penetrate the fabric, thereby increasing the UPF [30,34]. Kim and Jang [37] observed that UV/ozone treatment on meta-aramid films increased surface roughness at a nanoscale level but led to overall smoother, less fibrous surfaces due to the formation of oxidation products.

In addition, some of the construction properties of fabrics that affect UV resistance were also tested, namely density (Table 5) and thread diameter (Table 6) for the three samples, including mean values X and SD values. When selecting the samples, all three groups were considered and the samples were chosen according to maximum difference in UPF before and after Xe light exposure, (Sheath: para-aramid, Core: PA HT—difference 25.20); samples with medium difference (Sheath: meta-aramid, Core: - —difference 13.76), and samples with minimum difference (Sheath: PES Trevira CS, Core: PP—difference 0.50).

Table 5 shows that the density of the fabric sample with a para-aramid sheath and PA HT core slightly increases after Xe light exposure. This increase is accompanied by larger changes in yarn diameter, particularly in the weft yarn of the para-aramid/PA HT combination. In comparison, smaller changes in diameter are observed in the 100% meta-aramid sample, while minimal differences are seen in the fabric with a PES Trevira CS sheath and a PP core (Figure 4). This is consistent with the values of the UV results, as the structure of the yarn also changed during exposure, but not in the case of PES Trevira CS. As for yarns, UV exposure can lead to changes in diameter due to material degradation. The increase in diameter of para-aramid fibers after UV exposure is an interesting phenomenon. UV exposure causes the outer layers of the para-aramid fibers to degrade and oxidize, leading to the formation of a more amorphous structure. This structural change allows the fibers to absorb moisture and swell, which increases their diameter. The degradation of the fiber surface also leads to the release of internal stresses, further contributing to the increase in diameter. This phenomenon is commonly observed in high-performance fibers subjected to prolonged UV exposure [6]. However, the exact behavior depends on the specific type of yarn and its composition. Zhang et al. [34] investigated the effects of solar UV radiation (after 48 and 144 h) on the tensile strength and structural properties of Twaron 2000 para-aramid fibers. They concluded that UV radiation caused intense surface damage and chain scission, but the crystalline structure of the fibers remained unchanged.

**Figure 6:**
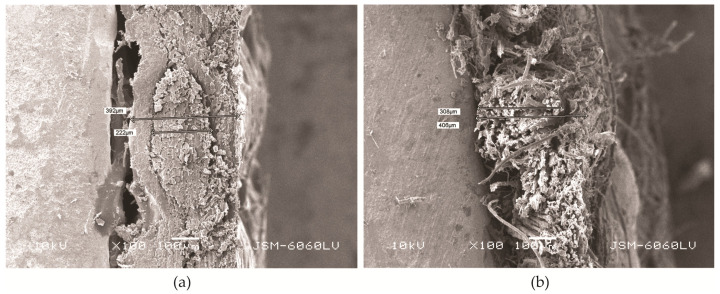
SEM images of cross-section of sheath: para-aramid, core: PA HT sample (**a**) before exposure, and (**b**) after exposure.

For the samples selected based on the maximum difference in UPF before and after Xe-light exposure, the sample with para-aramid sheath and PA HT core was used and a cross section was analyzed by SEM, as shown in Figure 6. The ordered structure of the weft yarn is visible before Xe-light exposure (Figure 6, left), while a disordered shape can be seen after Xe-light exposure (Figure 6, right), which explains the increase in yarn diameter. There is also a clear difference in the core and sheath diameter of the yarn before and after exposure. The deformation of the yarn is attributed to the Xe light and is related to the increase in diameter, which results in a more closed fabric surface and thus provides improved UV protection. However, this raises concerns about the mechanical properties of the material, particularly the strength of the samples.

### 3.5. Color Changes Due to UV Exposure

**Figure 7:**
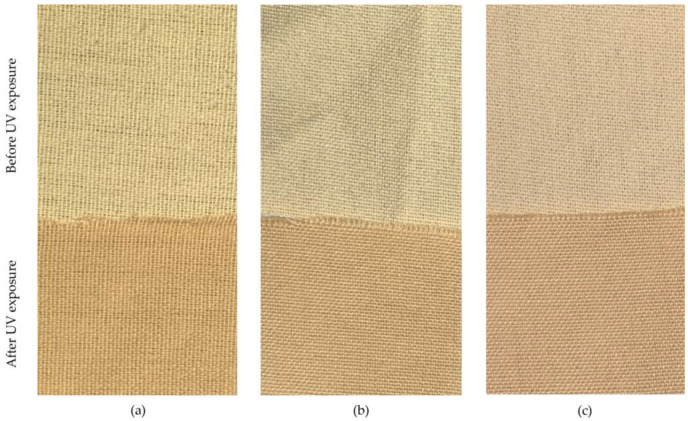
Color change on fabric samples after UV exposure: (**a**) Sheath: Para-aramid, Core: PA HT, (**b**) Sheath: Meta-aramid, Core: -, and (**c**) Sheath: PES Trevira CS, Core: PP.

Color changes due to UV exposure have been documented in various materials, including skin, wood, and fluorescent paints [38,39,40]. These findings align with the observed color change in para-aramid fibers (Figure 7), where UV exposure leads to a change from yellow to brownish-yellow, potentially improving UV absorption and protection. The color change from yellow to brownish-yellow in para-aramid fibers is caused by the formation of chromophores during UV exposure, as a result of oxidation and the breakdown of polymer chains. These chromophores absorb more UV radiation than the original yellow fibers, which enhances the material’s UV blocking capability. This color transition increases the fabric’s ability to absorb UV radiation, contributing to the improvement in UPF after exposure [6,30,34]. Nascimento et al. [29] attributed this color shift to the breakdown of polymeric chains and surface oxidation, which introduces carbonyl and hydroxyl groups. Similarly, Dong and Jang [28] observed that UV/ozone treatment resulted in increased oxygen content on the fiber surface, further leading to the formation of these functional groups, which contribute to the darkening of the fibers and enhance their UV-blocking capabilities.

## 4. Conclusions

This study extensively analyzed the UV protection properties of hybrid para-aramid woven fabrics with various special yarns in the core. The most important results indicate that fabrics a with para-aramid sheath exhibit the highest UPF values both before and after UV exposure. The UPF values increased significantly after exposure, which is due to the UV resistance of para-aramid fibers and the color change from yellow to brownish-yellow, enhancing UV absorption capabilities. Meta-aramid sheath fiber also shows improved UPF values after UV exposure, but less effective compared to para-aramid sheath.

The structural changes in meta-aramid fibers after UV exposure contributed to their UV-blocking ability, although not as much as para-aramid fibers. Polyester Trevira CS sheath fiber demonstrated the lowest UV protection among the fibers tested. Their UPF values did not increase significantly after UV exposure, indicating limited improvement in UV-blocking capabilities due to the lower UV resistance of polyester fibers. The choice of core materials plays a critical role in enhancing the UV protection properties. High-tenacity cores like Technora T240 and Dyneema SK75, when combined with para-aramid sheath, provided superior UV resistance. The surface of spun aramid fabrics became less hairy and smoother after UV exposure. This reduction in surface irregularities likely contributed to increased UPF due to a more compact and less porous structure. Additionally, the color change from yellow to brownish-yellow in para-aramid fibers after UV exposure positively impacted UPF values. This change in color is related to the formation of protective surface layers and enhanced UV absorbance characteristics.

This study confirms that the combination of high-crystallinity para-aramid fibers with suitable core materials significantly enhances UV protection. The formation of protective surface layers and the properties of para-aramid fibers contribute to increased UPF values after UV exposure. Further research could focus on long-term UV exposure and mechanical property assessments to fully understand the durability and protective capabilities of these fabrics.

## Figures and Tables

**Table 1:** The characteristics of the fabrics.

Sheath Fiber	Core Filament	Weight (g/m^2^)	Thickness (mm)	Fabric Density (kg/m^3^)
		X/SD	X/SD	
Para-aramid	-	183.16/2.060	0.53/0.013	345.58
	E-glass	168.04/2.166	0.52/0.005	323.15
	PES	183.57/5.778	0.50/0.011	367.14
	PA HT	184.80/4.750	0.51/0.007	362.35
	PP	185.64/1.964	0.54/0.019	343.78
	PES HT	178.23/2.657	0.52/0.019	342.75
	Technora T240	171.30/1.603	0.52/0.010	329.42
	Dyneema SK75	180.25/1.159	0.53/0.008	340.09
Meta-aramid	-	187.38/4.990	0.52/0.005	360.35
	E-glass	177.73/1.485	0.55/0.010	323.15
	PES	179.81/2.918	0.61/0.006	294.77
	PA HT	185.82/2.709	0.52/0.005	357.35
	PP	183.60/5.364	0.59/0.013	311.19
	PES HT	172.83/2.021	0.53/0.015	326.09
	Technora T240	186.38/3.030	0.55/0.015	338.87
	Dyneema SK75	187.58/2.997	0.57/0.007	329.09
PES Trevira CS	-	178.83/2.266	0.54/0.010	331.17
	E-glass	176.27/1.748	0.52/0.007	338.98
	PES	170.71/3.471	0.51/0.007	334.73
	PA HT	179.61/2.662	0.50/0.008	359.22
	PP	189.52/3.364	0.52/0.001	364.46
	PES HT	179.60/2.082	0.52/0.001	345.38
	Technora T240	178.88/2.756	0.52/0.007	344.00
	Dyneema SK75	191.02/9.960	0.54/0.010	353.74

**Table 2:** UPF, UVA, and UVB values before and after UV exposure.

Sheath Fiber Core Filament		UPF		UV A (315–400 nm)		UV B (290–315 nm)	
		Before	After	Before	After	Before	After
Para- aramid	-	48.71	49.75	1.88	1.90	2.13	1.99
	E-glass	32.38	34.73	2.89	2.82	3.22	2.90
	PES	47.46	34.33	1.78	2.87	2.04	2.98
	PA HT	53.63	79.53	1.79	1.22	1.97	1.26
	PP	57.88	72.61	1.86	1.36	1.96	1.38
	PES HT	42.75	51.60	2.27	1.92	2.53	1.94
	Technora T240	31.46	31.50	2.87	3.10	3.39	3.12
	Dyneema SK75	39.68	50.87	2.23	1.93	2.56	1.98
Meta- aramid	-	49.63	63.39	1.95	1.60	2.13	1.59
	E-glass	33.14	41.04	2.95	2.56	3.14	2.46
	PES	42.65	42.94	2.38	2.44	2.44	2.30
	PA HT	44.93	50.20	2.18	2.14	2.34	2.13
	PP	46.58	63.12	2.10	1.62	2.29	1.51
	PES HT	32.69	32.27	2.93	3.26	3.04	3.13
	Technora T240	53.77	65.41	1.79	1.60	2.02	1.53
	Dyneema SK75	42.09	52.60	2.40	2.09	2.50	1.91
PES Trevira CS	-	40.12	39.53	4.51	4.81	2.15	2.12
	E-glass	30.79	33.24	5.04	4.94	2.93	2.69
	PES	28.25	30.54	5.56	5.57	3.18	2.82
	PA HT	32.80	28.92	5.09	5.81	2.68	2.98
	PP	37.45	37.96	5.29	5.18	2.37	2.18
	PES HT	32.42	26.87	5.28	6.37	2.75	3.19
	Technora T240	29.61	29.43	4.84	4.93	3.19	3.06
	Dyneema SK75	39.33	41.59	4.74	4.66	2.15	2.05

**Table 3:** *p* values of the effect of material type on the UV (before/after exposure) and UVR (before/after exposure) properties and variance analysis of UV (before/after exposure) and UVR (before/after exposure) results.

		F					Sig.				
UV (before)		64.738					0.000 *				
UV (after)		76.543					0.000 *				
UVR (before)		91.864					0.000 *				
UVR (after)		59.198					0.000 *				
UV (before exposure)											
Code	N	Subset for Alpha = 0.05									
		1	2	3	4	5	6	7	8	9	
Meta-aramid/Technora	5	1.75									
Para-aramid/PA HT	5		1.81								
Para-aramid/PES	5		1.84								
Para-aramid/PP	5		1.85								
Para-aramid	5			1.94							
Meta-aramid	5			1.99							
Para-aramid/PES HT	5				2.14						
Meta-aramid/PP	5				2.24						
Meta-aramid/PA HT	5				2.25						
Meta-aramid/PES	5					2.40					
Para-aramid/Dyneema	5					2.43					
Meta-aramid/Dyneema	5					2.43					
Meta-aramid/PES HT	5						2.93				
Para-aramid/Technora	5						2.94				
Para-aramid/E-glass	5						2.97				
Meta-aramid/E-glass	5						3.00				
PES Trevira	5							3.96			
PES Trevira/Dyneema	5							4.13			
PES Trevira/Technora	5							4.46	4.46		
PES Trevira/PA HT	5								4.53		
PES Trevira/E-glass	5								4.55		
PES Trevira/PP	5								4.69		
PES Trevira/PES HT	5								4.72	4.72	
PES Trevira/PES	5									5.00	
Sig.		1.000	0.081	0.059	0.093	0.69	0.192	0.075	0.346	0.063	
UV (after exposure)											
Code	N	Subset for alpha = 0.05									
		1	2	3	4	5	6	7	8	9	10
Para-aramid/PA HT	5	1.23									
Para-aramid/PP	5	1.37									
Meta-aramid/PP	5		1.51								
Meta-aramid/Technora	5		1.53								
Meta-aramid	5		1.59								
Para-aramid	5			1.87							
Meta-aramid/Dyneema	5			1.91							
Para-aramid/PES HT	5			1.92							
Para-aramid/Dyneema	5			1.94							
Meta-aramid/PA HT	5				2.13						
Meta-aramid/PES	5				2.30						
Meta-aramid/E-glass	5				2.46						
Para-aramid/E-glass	5					2.84					
Para-aramid/PES	5					2.90	2.90				
Para-aramid/Technora	5						3.07				
Meta-aramid/PES HT	5						3.13				
PES Trevira/Dyneema	5							4.05			
PES Trevira	5							4.18			
PES Trevira/E-glass	5								4.38		
PES Trevira/PP	5								4.48		
PES Trevira/Technora	5								4.50		
PES Trevira/PES	5									4.93	
PES Trevira/PA HT	5									5.15	
PES Trevira/PES HT	5										5.63
Sig.		0.102	0.118	0.109	0.084	0.058	0.071	0.059	0.083	0.062	1.000
UVR (before exposure)											
Code	N	Subset for alpha = 0.05									
		1	2	3	4	5	6	7	8	9	
Para-aramid	5	8.27									
Para-aramid/E-glass	5	8.48	8.48								
Para-aramid/Technora	5	8.54	8.54								
Para-aramid/PES HT	5	8.56	8.56								
Para-aramid/PP	5	8.58	8.58								
Para-aramid/PES	5		8.67								
Para-aramid/Dyneema	5		8.76								
Para-aramid/PA HT	5		8.84								
Meta-aramid/Technora	5			10.88							
Meta-aramid/PP	5			11.30	11.30						
Meta-aramid/PES HT	5			11.32	11.32						
Meta-aramid/PES	5				11.67						
Meta-aramid/E-glass	5				11.98	11.98					
Meta-aramid/PA HT	5					12.12					
Meta-aramid/Dyneema	5					12.17					
Meta-aramid	5					12.35					
PES Trevira/Technora	5						15.44				
PES Trevira	5							16.33			
PES Trevira/PA HT	5							16.63			
PES Trevira/Dyneema	5							16.73			
PES Trevira/PES HT	5							16.90	16.90		
PES Trevira/PP	5								17.37		
PES Trevira/E-glass	5								17.80		
PES Trevira/PES	5									18.52	
Sig.		0.569	0.294	0.759	0.387	0.256	1.000	0.109	0.165	1.000	
UVR (after exposure)											
Code	N	Subset for alpha = 0.05									
		1	2	3	4	5	6	7	8	9	
Para-aramid	5	6.10									
Para-aramid/Technora	5	6.12									
Para-aramid/PES HT	5	6.31									
Para-aramid/E-glass	5	6.32	6.32								
Para-aramid/PES	5		6.38	6.38							
Para-aramid/Dyneema	5		6.39	6.39							
Para-aramid/PP	5			6.58							
Para-aramid/PA HT	5			6.58							
Meta-aramid/Technora	5			6.92	6.92						
Meta-aramid/PES	5				7.09	7.09					
Meta-aramid/PES HT	5					7.22					
Meta-aramid/E-glass	5					7.24					
Meta-aramid/PP	5					7.30					
Meta-aramid	5					7.38					
Meta-aramid/PA HT	5					7.81	7.81				
Meta-aramid/Dyneema	5						8.21				
PES Trevira/Technora	5							14.76			
PES Trevira/E-glass	5							15.84			
PES Trevira/PA HT	5							16.09	16.09		
PES Trevira/PES	5								16.71		
PES Trevira/PES HT	5								16.85		
PES Trevira	5								16.95		
PES Trevira/PP	5									17.54	
PES Trevira/Dyneema	5									17.78	
Sig.		0.843	0.437	0.398	0.076	0.254	0.112	0.079	0.163	0.103	

* Significant at 0.05 level.

**Table 4:** UV R (%) and UV A (%) values before and after UV exposure.

Sheath Fiber	Core Filament	UV R (%)		UV A (%)	
		Before	After	Before	After
		X/SD	X/SD		
Para-aramid	-	8.27/0.031	6.10/0.081	89.78	92.03
	E-glass	8.48/0.156	6.32/0.127	88.55	90.85
	PES	8.67/0.055	6.38/0.193	89.49	90.72
	PA HT	8.84/0.094	6.58/0.054	89.35	92.20
	PP	8.58/0.176	6.58/0.182	89.57	92.05
	PES HT	8.56/0.141	6.31/0.027	91.44	91.76
	Technora T240	8.54/0.255	6.12/0.152	88.52	90.82
	Dyneema SK75	8.76/0.269	6.39/0.068	88.81	91.66
Meta-aramid	-	12.35/0.214	7.38/0.078	85.66	90.95
	E-glass	11.98/0.350	7.24/0.077	85.02	90.22
	PES	11.67/0.151	7.09/0.155	85.93	90.44
	PA HT	12.12/0.305	7.81/0.119	85.63	89.99
	PP	11.30/0.334	7.30/0.060	86.46	91.15
	PES HT	11.32/0.430	7.22/0.091	85.74	89.55
	Technora T240	10.88/0.384	6.92/0.106	87.37	91.49
	Dyneema SK75	12.17/0.268	8.21/0.046	85.40	89.73
PES Trevira CS	-	16.33/0.480	16.95/0.240	79.71	78.86
	E-glass	17.80/0.423	15.84/0.201	77.65	79.78
	PES	18.52/0.629	16.71/0.217	76.48	78.35
	PA HT	16.63/0.370	16.09/0.161	78.84	78.76
	PP	17.37/0.250	17.54/0.219	77.95	77.97
	PES HT	16.90/0.390	16.85/0.213	78.38	77.52
	Technora T240	15.44/0.083	14.76/0.340	80.10	80.74
	Dyneema SK75	16.73/0.493	17.78/0.014	79.14	78.18

**Table 5:** Density of threads before and after exposure.

Samples	Density (Threads/cm)			
	Warp		Weft	
	Before	After	Before	After
	X/SD	X/SD	X/SD	X/SD
Sheath: Para-aramid, Core: PA HT	15.94/0.000	16.06/0.577	20.42/0.707	21.10/0.577
Sheath: Meta-aramid, Core: -	15.39/0.000	15.43/0.000	21.32/0.000	21.32/0.000
Sheath: PES Trevira CS, Core: PP	15.87/0.000	15.69/1.414	22.22/0.707	21.53/0.707

**Table 6:** Diameter of threads before and after exposure.

Samples	Diameter (mm)			
	Warp		Weft	
	Before	After	Before	After
	X/SD	X/SD	X/SD	X/SD
Sheath: Para-aramid, Core: PA HT	0.43/0.033	0.45/0.028	0.38/0.051	0.44/0.047
Sheath: Meta-aramid, Core: -	0.41/0.021	0.43/0.029	0.37/0.030	0.39/0.029
Sheath: PES Trevira CS, Core: PP	0.42/0.032	0.42/0.040	0.36/0.026	0.37/0.034

## Data Availability

The data presented in this study are available on request from the corresponding author.
